# Supplementary material for: Measurement of Cellular Immune Response to Viral Infection and Vaccination
Source: Front Immunol. 2020 Oct 19;11:575074. doi: 10.3389/fimmu.2020.575074 (PMC7604353; doi:10.3389/fimmu.2020.575074)
Supplement: Supplementary file 1 [file DataSheet_1.pdf]

SUPPLEMENTARY INFORMATION

Development of JAK/STAT1/2 and JAK-STAT3 pathway tests, Supplementary figures

Figure S1

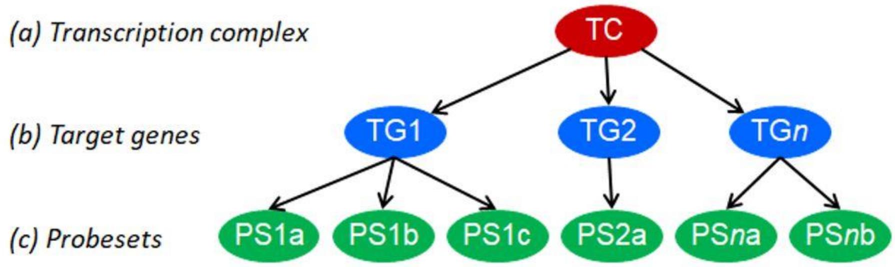

Figure S1. Knowledge-based Bayesian computational pathway model. The Bayesian network structure used as a basis for our modeling approach shown as a simplified model of the transcriptional program of a cellular signal transduction pathway, consisting of three types of nodes: transcription factor complex (TC), target gene (TG), and microarray probe sets (PS) corresponding to target genes.

With permission [1].

Figure S2A

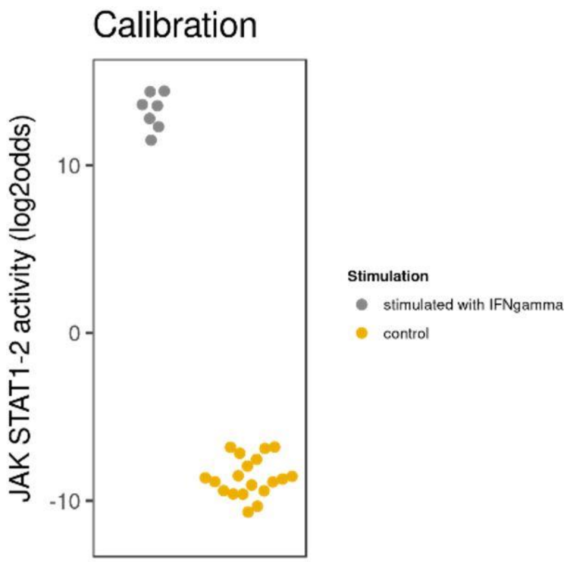

Figure S2B

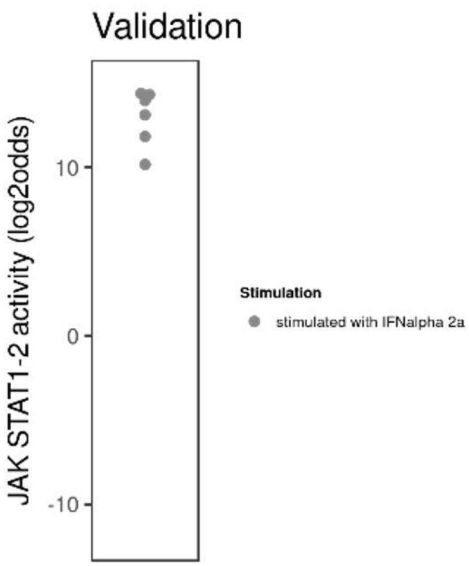

Figure S2C

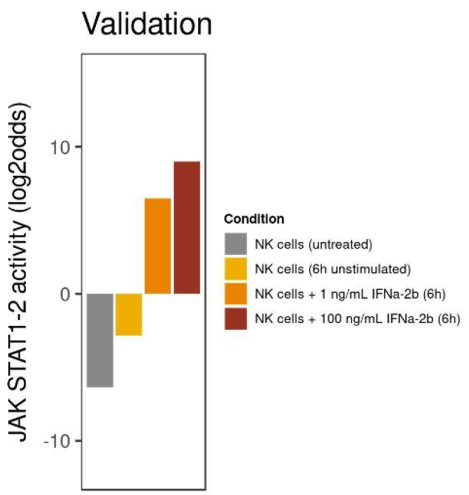

Figure S2D

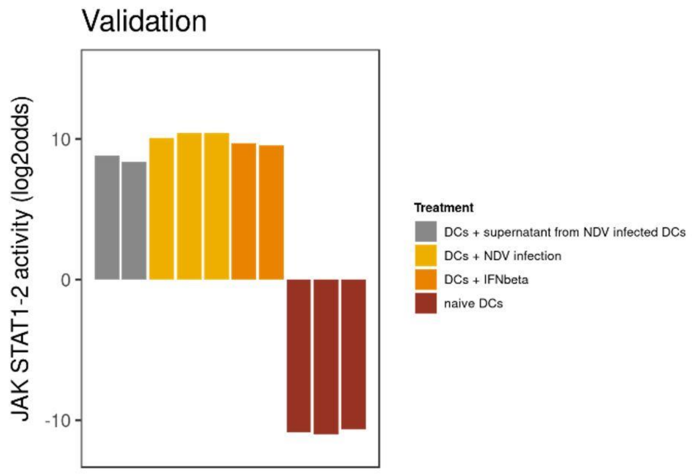

Figure S2E

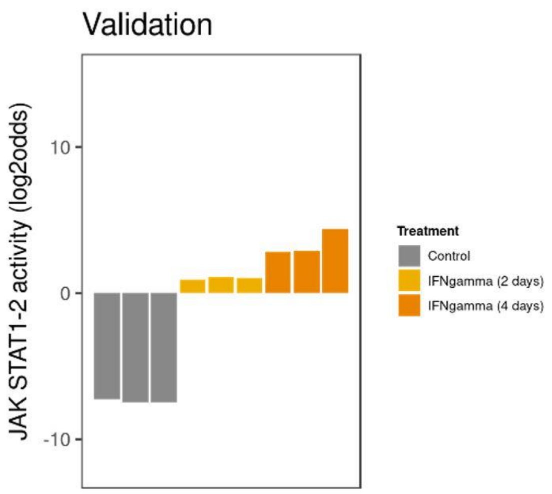

Figure S2F

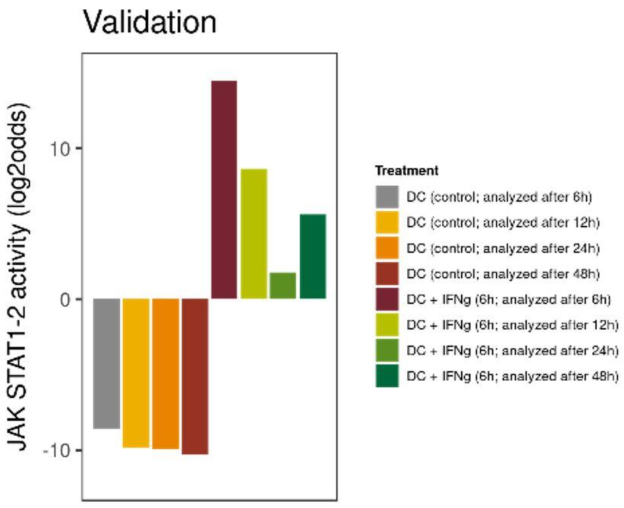

Figure S2G

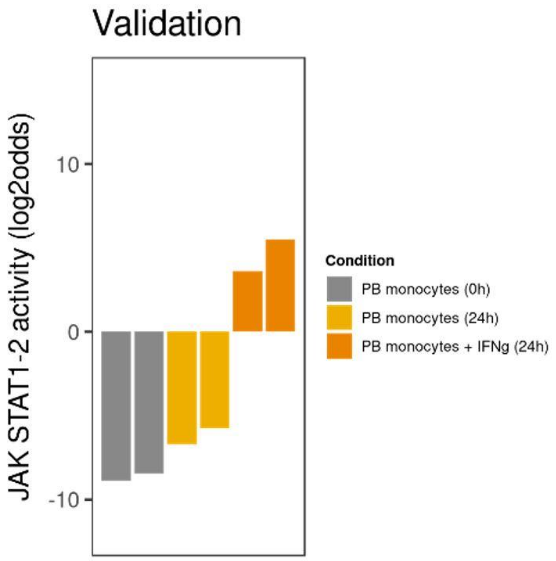

Figure S2. Calibration and biological validation of the JAK-STAT 1/2 model.

A: Results on microarray data from GSE38351 [2]: calibration of JAK-STAT1/2 pathway model with peripheral blood (PB) monocytes stimulated with 100 ng/mL interferon type II (IFNγ; n=7; active pathway) for 1.5 hours and unstimulated (n=19; inactive pathway).

B-D. Validation of JAK-STAT1/2 pathway model for IFN type I induced pathway activity.

B. Results on microarray data from GSE38351 [2]: peripheral blood monocytes stimulated for 1.5 hrs with IFNα2a (n=7).

C. Results on microarray data from GSE15743 [3]: peripheral natural killer (NK) cells, untreated, cultured for 6 hours without stimulation, treated for 6 hours with 1 ng/mL recombinant IFNα-2b (low interferon α) and treated with 100 ng/mL recombinant IFNα-2b (high interferon α).

D. Results on microarray data from GSE52081 [4]: dendritic cells, exposed to supernatant of Newcastle Disease Virus (NDV)-infected cells, infected with NDV, treated with IFNβ or kept as naïve dendritic cells.

E-G: Validation of the JAK-STAT1/2 pathway model for IFN type II-induced pathway activity

E: Results on microarray data from GSE58096 [5]: THP1 human monocyte-like cells untreated or treated with IFNγ for 2 or 4 days.

F: Results on microarray data from GSE11327 [6]: immature monocyte-derived dendritic cells untreated or matured with 30 ng/mL lipopolysaccharide (LPS) and 1000 U/mL IFNγ for 6 hours, and analyzed at 6, 12, 24 and 48 hours

G: Results on microarray data from GSE11864 [7]: peripheral blood monocytes (from 2 donors) were directly analyzed or cultured in the absence or presence of 100 U/mL IFNγ for 24 hours.

The pathway activity score is presented on a log2 odds scale. Each dot represents one sample. In case only a few samples needed presentation, bar plots are used instead of dot blots, each bar representing one sample

Figure S3A

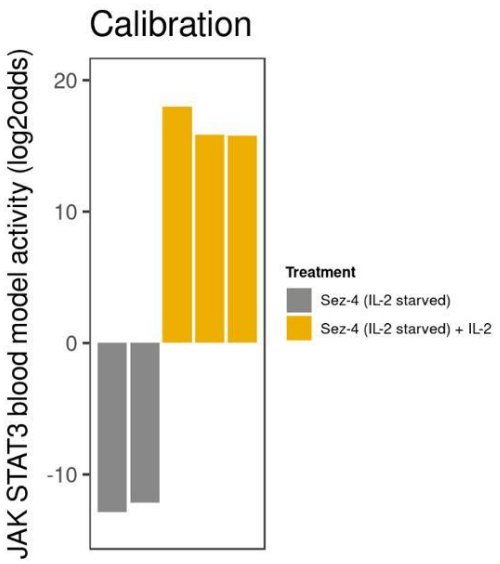

Figure S3B

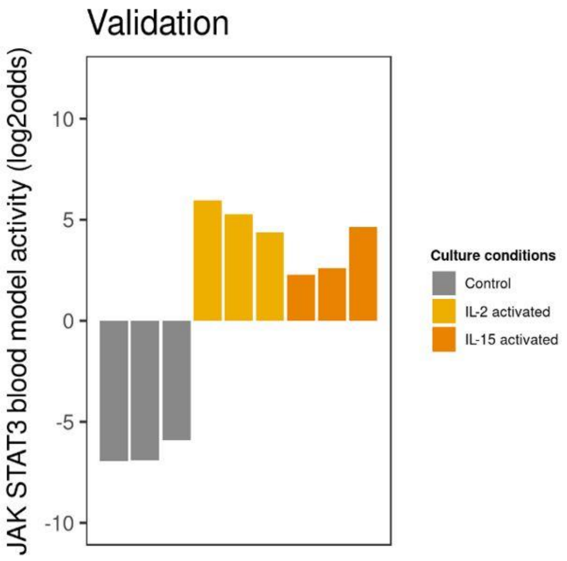

Figure S3C

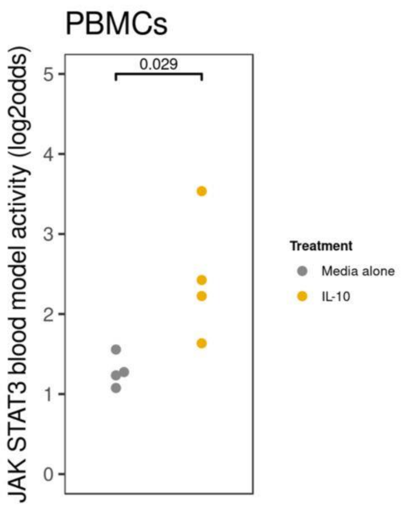

Figure S3D

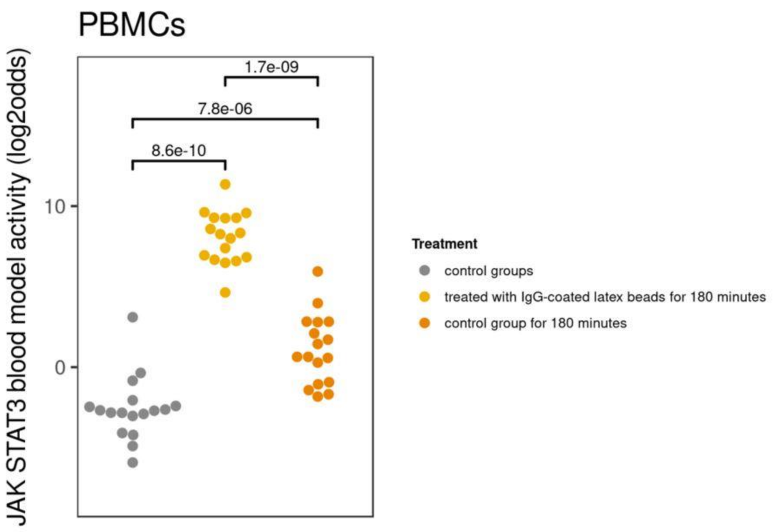

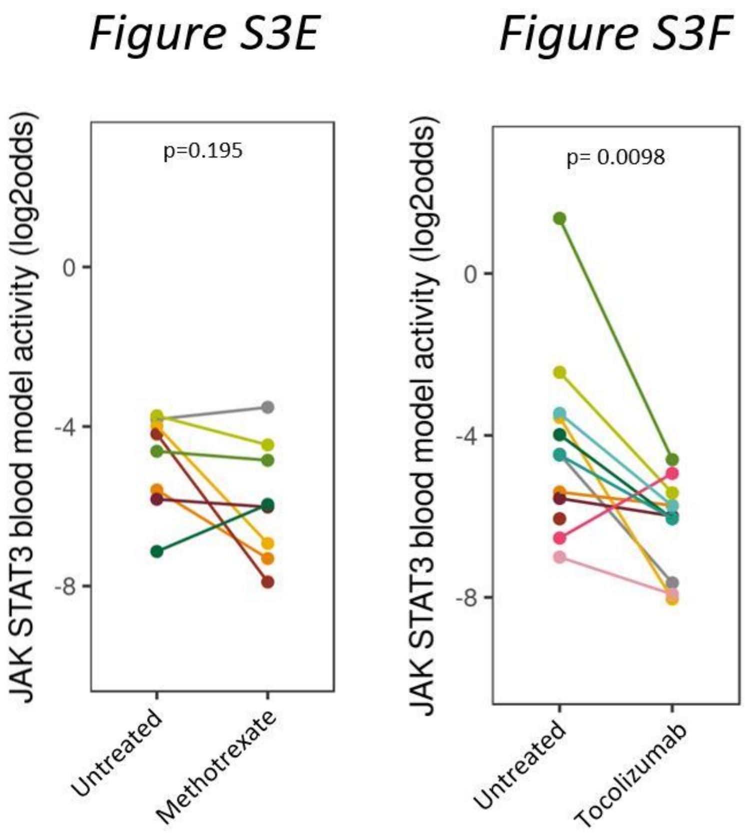

Figure S3. Calibration and biological validation of JAK-STAT3 pathway model for use on blood cells (JAK-STAT3-B model).

A: Results on microarray data from GSE8687 [8]: calibration of the JAK-STAT3-B pathway model with CD4+ T cells derived from leukemic (Sezary) cells of a patient with a cutaneous T-cell lymphoma. Cells were starved of Interleukin-2 (IL-2; n=2; inactive pathway) or cultured with IL-2 (n=3; active pathway).

B: Results on microarray data from GSE8685 [8]: validation of the JAK-STAT3-B model on independent samples from Sez-4 T cell lymphoma cells that were cultured 16 hours in absence of IL-2 (IL-2 starved) and subsequently treated with 200 U IL-2, 20 ng/mL IL-15 or vehicle (control) for 4 hours.

C. Results on microarray data from GSE43700 [9]: PBMCs from blood of healthy human donors (n=4), stimulated by IL-10 (R&D Systems) 10ng/ml or vehicle for 24 hours. Two-sided Wilcoxon signed-rank statistical tests performed, p-values are indicated in de figures.

D. Results on microarray data from GSE8507 [10]: PBMCSs measured directly, or stimulated with IgG-coated latex beads or control for 180 minutes. Two-sided Wilcoxon signed-rank statistical tests performed, p-values are indicated in de figures.

E-F. Results on microarray data from GSE45867 [11]: knee synovial biopsies taken from patients with RA, before (untreated) and after 12 weeks of treatment with or methotrexate (treatment naive pts) (E) or Tocilizumab (IL-6 inhibitor) (F). Tocilizumab was clinically more effective. Paired patient samples, analyzed with paired t-test: comparison between untreated and Tocilizumab (n=11), p=0.0098; between untreated and methotrexate (n=8): p=0.195 (ns).

The pathway activity score is presented on a log2 odds scale. Each dot represents one sample. In case only a few samples needed presentation, bar plots are used instead of dot blots, each bar representing one sample.

Supplementary Methods

Selection of target genes for Bayesian models for JAK-STAT1/2 and JAK-STAT3 pathway models

For each putative target gene of a pathway-associated transcription factor, evidence was assessed for the presence of a binding element in the gene promoter region, functionality of the binding element (e.g., in promoter-luciferase experiments), binding of the transcription factor to the

respective response/enhancer element *in vivo* (e.g., using ChIPseq) and/or *in vitro* (using Electrophoretic Mobility Shift Assay, EMSA), and differential expression with pathway activation. Gene selection was also based on consistency of evidence as reported by multiple research groups for multiple cell/tissue types. Around 20 genes per pathway were selected, which is high enough to enable robustness and sensitivity of the pathway assay, while allowing for maximal specificity. Because the target genes only function to read out transcription factor activity, they were selected based on evidence for reproducible and specific transcription factor-induced transactivation across various cell types, and not based on function of encoded proteins. Probesets on the Affymetrix HG-U133Plus2.0 microarray associated with the target genes were selected based on the Bioconductor package available in R and manual curation using the latest information available on the UCSC Genome Browser (www.genome.ucsc.edu) [12].

Supplementary Tables

Table S1. List with target genes used in the Bayesian models to develop JAK-STAT1/2 and JAK-STAT3 signal transduction pathway tests

JAK-STAT1/2 signaling pathway: BID, GNAZ, IRF1, IRF7, IRF8, IRF9, LGALS1, NCF4, NFAM1, OAS1, PDCD1, RAB36, RBX1, RFPL3, SAMM50, SMARCB1, SSTR3, ST13, STAT1, TRMT1, UFD1L, USP18, ZNRF3.

JAK-STAT3 signaling pathway: AKT1, BCL2, BCL2L1, BIRC5, CCND1, CD274, CDKN1A, CRP, FGF2, FOS, FSCN1, FSCN2, FSCN3, HIF1A, HSP90AA1, HSP90AB1, HSP90B1,

HSPA1A, HSPA1B, ICAM1, IFNG, IL10, JUNB, MCL1, MMP1, MMP3, MMP9, MUC1, MYC, NOS2, POU2F1, PTGS2, SAA1, STAT1, TIMP1, TNFRSF1B, TWIST1, VIM, ZEB1.

Table S2. References for target gene selection

JAK-STAT1/2 pathway

[13],[14],[15],[16],[17],[18],[19],[20],[21],[22],[23]

JAK-STAT3 pathway

[24],[25],[26],[27]

Table S3 JAK-STAT pathway activity scores in PBMC samples of health individuals

S3A. Healthy individual samples from datasets GSE34205 [28] and GSE13486 [29], separate groups.

GSE dataset	Female (F) / male (M)	JAK-STAT1/2 pathway activity score (*log2odds*)<br>*Mean*	JAK-STAT1/2 pathway activity score (*log2odds*)<br>*Standard deviation*	JAK-STAT3 pathway activity score (*log2odds*)<br>*Mean*	JAK-STAT3 pathway activity score (*log2odds*)<br>*Standard deviation*
GSE34205	F	-5.35	3.13	-7.49	1.15
GSE34205	M	-5.84	2.74	-8.18	1.91
GSE13486	F+M	-6.50	2.56	-7.73	1.79

S3B. Healthy individual samples from datasets GSE34205 [28] and GSE13486 [29] were combined.

**GSE dataset**	**Female (F) / male (M)**	**JAK-STAT1/2 pathway activity score (*log2odds*)**<br><br>***Mean***	**JAK-STAT1/2 pathway activity score (*log2odds*)**<br><br>***Standard deviation***	**JAK-STAT3 pathway activity score (*log2odds*)**<br><br>***Mean***	**JAK-STAT3 pathway activity score (*log2odds*)**<br><br>***Standard deviation***
GSE34205<br>GSE13486	F+M	-5.95	2.77	-7.86	1.72

Supplementary references

[1] W. Verhaegh *et al.*, “Selection of personalized patient therapy through the use of knowledge-based computational models that identify tumor-driving signal transduction pathways,” *Cancer Res.*, vol. 74, no. 11, pp. 2936–2945, Jun. 2014.

[2] A. Menßen *et al.*, “SiPaGene: A new repository for instant online retrieval, sharing and meta-analyses of GeneChip® expression data,” *BMC Genomics*, vol. 10, no. 1, p. 98, Mar. 2009.

[3] K. A. Stegmann *et al.*, “Interferon-α-Induced TRAIL on Natural Killer Cells Is Associated With Control of Hepatitis C Virus Infection,” *Gastroenterology*, vol. 138, no. 5, pp. 1885-1897.e10, May 2010.

[4] B. M. Hartmann, N. Marjanovic, G. Nudelman, T. M. Moran, and S. C. Sealfon, “Combinatorial Cytokine Code Generates Anti-Viral State in Dendritic Cells,” *Front. Immunol.*, vol. 5, no. FEB, p. 73, Feb. 2014.

[5] J. S. Leu *et al.*, “SP110b controls host immunity and susceptibility to tuberculosis,” *Am. J. Respir. Crit. Care Med.*, vol. 195, no. 3, pp. 369–382, Feb. 2017.

[6] K. Soukup *et al.*, “The MAPK-Activated Kinase MK2 Attenuates Dendritic Cell–Mediated Th1 Differentiation and Autoimmune Encephalomyelitis,” *J. Immunol.*, vol. 195, no. 2, pp. 541–552, Jul. 2015.

[7] X. Hu *et al.*, “Integrated Regulation of Toll-like Receptor Responses by Notch and Interferon-γ Pathways,” *Immunity*, vol. 29, no. 5, pp. 691–703, Nov. 2008.

[8] M. Marzec *et al.*, “Differential effects of interleukin-2 and interleukin-15 versus interleukin-21 on CD4+ cutaneous T-cell lymphoma cells,” *Cancer Res.*, vol. 68, no. 4, pp. 1083–1091, Feb. 2008.

[9] R. M. B. Teles *et al.*, “Type I interferon suppresses type II interferon-triggered human anti-mycobacterial responses,” *Science (80-.).*, vol. 339, no. 6126, pp. 1448–1453, Mar. 2013.

[10] S. M. Holland *et al.*, “*STAT3* Mutations in the Hyper-IgE Syndrome,” *N. Engl. J. Med.*, vol. 357, no. 16, pp. 1608–1619, Oct. 2007.

[11] B. R. Lauwerys *et al.*, “Heterogeneity of Synovial Molecular Patterns in Patients with Arthritis,” *PLoS One*, vol. 10, no. 4, p. e0122104, Apr. 2015.

[12] W. Huber *et al.*, “Orchestrating high-throughput genomic analysis with Bioconductor,” *Nat. Methods*, vol. 12, no. 2, pp. 115–121, Jan. 2015.

[13] S. E. Hartman *et al.*, “Global changes in STAT target selection and transcription regulation upon interferon treatments,” *Genes Dev.*, vol. 19, no. 24, pp. 2953–2968, Dec. 2005.

[14] I. Wiesauer, C. Gaumannmüller, I. Steinparzer, B. Strobl, and P. Kovarik, “Promoter Occupancy of STAT1 in Interferon Responses Is Regulated by Processive Transcription,” *Mol. Cell. Biol.*, vol. 35, no. 4, pp. 716–727, Feb. 2015.

[15] A. Kumatori, D. Yang, S. Suzuki, and M. Nakamura, “Cooperation of STAT-1 and IRF-1 in interferon-γ-induced transcription of the gp91phox gene,” *J. Biol. Chem.*, vol. 277, no. 11, pp. 9103–9111, Mar. 2002.

[16] B. Basham *et al.*, "In vivo identification of novel STAT5 target genes," *Nucleic Acids Res.*, vol. 36, no. 11, pp. 3802–3818, Jun. 2008.

[17] T. J. J. Wood *et al.*, "Specificity of transcription enhancement via the STAT responsive element in the serine protease inhibitor 2.1 promoter," *Mol. Cell. Endocrinol.*, vol. 130, no. 1–2, pp. 69–81, Jun. 1997.

[18] T. J. J. Wood *et al.*, "Mediation of growth hormone-dependent transcriptional activation by mammary gland factor/stat 5," *J. Biol. Chem.*, vol. 270, no. 16, pp. 9448–9453, 1995.

[19] N. Kiuchi *et al.*, "STAT3 is required for the gp130-mediated full activation of the c-myc gene," *J. Exp. Med.*, vol. 189, no. 1, pp. 63–73, Jan. 1999.

[20] B. Emanuelli, P. Peraldi, C. Filloux, D. Sawka-Verhelle, D. Hilton, and E. Van Obberghen, "SOCS-3 is an insulin-induced negative regulator of insulin signaling," *J. Biol. Chem.*, vol. 275, no. 21, pp. 15985–15991, May 2000.

[21] H. Takatori *et al.*, "Stat5a Inhibits IL-12-Induced Th1 Cell Differentiation through the Induction of Suppressor of Cytokine Signaling 3 Expression," *J. Immunol.*, vol. 174, no. 7, pp. 4105–4112, Apr. 2005.

[22] H. W. Lo *et al.*, "Epidermal growth factor receptor cooperates with signal transducer and activator of transcription 3 to induce epithelial-mesenchymal transition in cancer cells via up-regulation of TWIST gene expression," *Cancer Res.*, vol. 67, no. 19, pp. 9066–9076, Oct. 2007.

[23] E. Yang, L. Lerner, D. Besser, and J. E. Darnell, "Independent and cooperative activation of chromosomal c-fos promoter by STAT3," *J. Biol. Chem.*, vol. 278, no. 18, pp. 15794–15799, May 2003.

[24] R. L. Carpenter and H. W. Lo, "STAT3 target genes relevant to human cancers," *Cancers*, vol. 6, no. 2. MDPI AG, pp. 897–925, 2014.

[25] H. Yu, D. Pardoll, and R. Jove, “STATs in cancer inflammation and immunity: A leading role for STAT3,” *Nature Reviews Cancer*, vol. 9, no. 11. Nat Rev Cancer, pp. 798–809, Nov-2009.

[26] M. Snyder, X. Y. Huang, and J. Jillian Zhang, “Identification of novel direct stat3 target genes for control of growth and differentiation,” *J. Biol. Chem.*, vol. 283, no. 7, pp. 3791–3798, Feb. 2008.

[27] S. Desrivières, C. Kunz, I. Barash, V. Vafaizadeh, C. Borghouts, and B. Groner, “The biological functions of the versatile transcription factors STAT3 and STAT5 and new strategies for their targeted inhibition,” *Journal of Mammary Gland Biology and Neoplasia*, vol. 11, no. 1. J Mammary Gland Biol Neoplasia, pp. 75–87, Jan-2006.

[28] I. Ioannidis *et al.*, “Plasticity and Virus Specificity of the Airway Epithelial Cell Immune Response during Respiratory Virus Infection,” *J. Virol.*, vol. 86, no. 10, pp. 5422–5436, May 2012.

[29] T. D. Querec *et al.*, “Systems biology approach predicts immunogenicity of the yellow fever vaccine in humans,” *Nat. Immunol.*, vol. 10, no. 1, pp. 116–125, Nov. 2009.
